# Heavy Metals Removing from Municipal Solid Waste Incineration Fly Ashes by Electric Field-Enhanced Washing

**DOI:** 10.3390/ma13030793

**Published:** 2020-02-10

**Authors:** Yang Tian, Rong Wang, Zhenggang Luo, Rui Wang, Feihua Yang, Zhaojia Wang, Jiancheng Shu, Mengjun Chen

**Affiliations:** 1 State Key Laboratory of Solid Waste Reuse for Building Materials, Beijing Building Materials Academy of Sciences Research, Beijing 100041, China; tianyang941005@gmail.com (Y.T.); chyangfeihua@126.com (F.Y.); zhaojiaw@bbma.com.cn (Z.W.); 2 Key Laboratory of Solid Waste Treatment and Resource Recycle, Ministry of Education, Southwest University of Science and Technology, Mianyang 621010, China; wr276432218@163.com (R.W.); luo787424395@163.com (Z.L.); wangrui6393@163.com (R.W.)

**Keywords:** MSWI fly ash, electric field, heavy metals, harmless

## Abstract

Municipal solid waste incineration (MSWI) fly ash contains chlorides, heavy metals, and organic pollutants, which requires appropriate disposal to eliminate this risk. In this study, the effects of agents on heavy metals removal from MSWI fly ash by electric field-enhanced washing were systematically studied. The results show that when these fly ashes were washed at a current density of 35 mA/cm^2^, polarity switching frequency of 40 Hz, Ethylenediaminetetraacetic acid (EDTA) dosage of 0.5 mol/L, and a pH of 2 for 4 h, almost all of the Cd and Ni could be were removed, with a removal efficiency of 100.00% and 99.59%, respectively. Meanwhile, it also shows a significant effect on Cu and Zn, with a removal efficiency higher than 85%. After washing, the results of the sequential extraction procedure showed that the residual forms of Pb, Cu, Zn, Cd, Ni, and As increased obviously. According to GB5085.3-2007, the toxicity of the treated MSWI fly ash were below their thresholds of 5 and 1 mg/L for Pb and Cd, respectively. Thus, a novel technology for heavy metals removal from MSWI fly ash is proposed.

## 1. Introduction

As the sharp increasing growth of economy and the significant acceleration of urbanization, about two-thirds of Chinese cities are suffering from “garbage siege” [1]. Statistically, in 2017, the municipal solid waste (MSW) produced by 202 large and medium-sized cities nationwide amounted to 20 million tons, increasing by approximately 8–10% annually. Incineration is widely used to deal with MSW, owing to its advantages of good capacity reduction, high degree of harmlessness, and high energy efficiency [2]. It is reported that China has built and operated 220 MSWI plants in large and medium-sized cities over the past decades, with an incineration capacity of 219,000 tons/day, 62 million tons per year, accounting for 26.9% of China’s annual waste disposal volume. However, in the process of incineration, about 3–5% municipal solid waste incineration (MSWI) fly ash will be produced [3]. Due to its fine size and large specific surface area, MSWI fly ash is prone to enrich not only heavy metals in high concentrations, but also dioxins, which are highly toxic and powerful carcinogenic organic pollutants, thus posing serious risk to the environment and human beings [4]. Therefore, MSWI fly ash has been classified as a hazardous waste and requires effective treatment prior to landfill or comprehensive utilization. 

MSWI fly ash disposal are mainly “solidification/stabilization + landfill”. Various treatment techniques, including cement solidification, chemical stabilization, and vitrification, etc., must be adopted before landfill to alleviate the toxicity. Cementitious materials, such as cement [5], asphalt [6], or stabilizing chemicals [7], are mixed and reacted with MSWI fly ash, and heavy metals are wrapped into hard solidified bodies. Solidification/stabilization, as a mature technology, has become the dominant method for the disposal of MSWI fly ash in most countries. However, solidification results in an increase of 1.5–2.0 times in final landfill volume and 30% in mass. The long-term environmental safety assessment of treated MSWI fly ash needs to be further verified [8]. 

Generally, it is significant to pre-treat the MSWI fly ash before hazard-free treatment or recycling, for mitigating the corrosion of disposal equipment or the adverse effect on the quality of resource products. With the popularization and application of electrochemical technology, substantial progress has been made: it has gradually expanded from soil remediation [9] to clay [10], copper tailings [11], electrolytic manganese residue [12], converter vanadium slag [13], refractory ores [14], and refractory gold ores [15]. Meanwhile, electrons could also be used as “cleaners” under a low-intensity electric field [16]. As a highly-porous hazardous waste, MSWI fly ash contains a large number of micropores, which is beneficial to the moisture transport in the reaction system. The large amount of metal ions contained in the micropores is also advantageous for improving the ion-exchange capacity of MSWI fly ash. Therefore, the electrochemical technique is thought to be an effective method to remove heavy metals from MSWI fly ash. To improve this removal efficiency, assistant agents were adopted since they could form a stable compound with heavy metals contained in MSWI fly ash, favoring to be removed [17]. In addition, such an assistant agent must be mobilized by an electric field such as Ethylenediaminetetraacetic acid (EDTA), which was applied in contaminated soil and heavy metals waste water [18,19].

Thus, four pivotal parameters of pH, current density, extraction time, and polarity switching frequency for heavy metals removal from MSWI fly ashes by electric enhanced washing were studied. Meanwhile, the effect of EDTA during this washing process was also discussed. The results show that EDTA-assisted electric washing could effectively remove heavy metals from MSWI fly ashes, providing a perspective approach for MSWI removing, while also bringing a new application for electrochemistry.

## 2. Materials and Methods

### 2.1. Materials 

MSWI fly ash used was collected from a certified MSWI plant in Beijing, China, under the bag filter, which was first dried to a constant weight at 80 °C. The chemical reagents such as NaOH, H_2_SO_4_, HNO_3_, HCl, HF, CH_3_COOH, HONH_3_Cl, H_2_O_2_, CH_3_COONH_4_, and EDTA used in this study were of analytical grade expect specific claim. Deionized water was provided by the Water Purification System (Advantage A10, Millipore, Burlington, MA, USA), and all experiments were carried out at room temperature.

### 2.2. Electric Enhanced Washing

**Figure 1:**
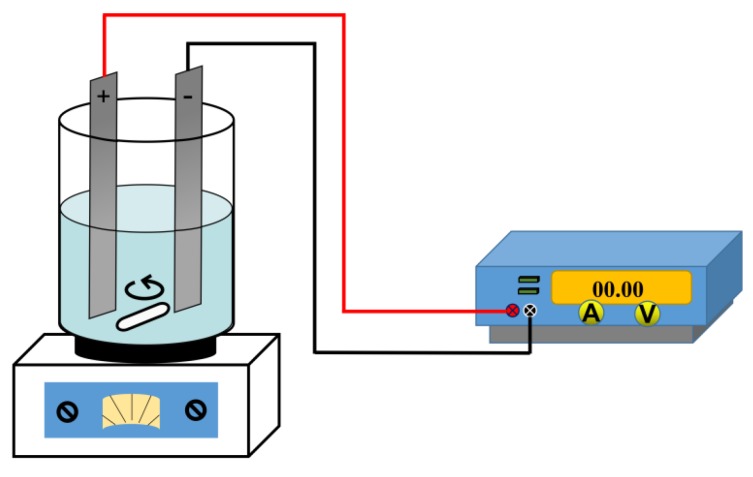
The experimental apparatus of enhanced extracted by electric field.

The experimental apparatus used in this study is shown in Figure 1. The reactor used in the experiment was a 500 mL beaker, and the anode and cathode were graphite plates (Qingdao BaoFeng graphite products Co., Ltd., Qingdao, China), having a surface area of 45 cm^2^ (4.5 × 10 cm). The two were fixed at a distance of 10 mm. For a typical experiment, 20 g of MSWI fly ash sample was added into the beaker and blended with 0.5 mol/L EDTA, while the liquid-to-solid (L/S) ratio was fixed at 15 mL/g. In addition, a direct current power supply (KRT-3005, Shenzhen JinRang electronic technology Co. Ltd., Shenzhen, China) with a current range of 0–3 A and voltage of 0–30 V was used to provide the electric field. The beaker was placed on a thermostatic magnetic stirrer (HJ-6A, Chanzhou Ronghua instrument manufacturing Co. LTD, Chanzhou, China), stirring at an appropriate stirring speed for a set time. During this process, pH value of the reaction system was monitored by a pH meter timely which was kept at a constant value by HNO_3_ or NaOH. After extracting, the supernatant was separated from the residue by vacuum filtration and determined by Inductively Coupled Plasma Optical Emission Spectrometer (ICP-OES, Optima 8300, PerkinElmer, Waltham, MA, USA). The remaining residue was dried at 65 °C for further analysis. The removal efficiency of heavy metals in MSWI fly ash is calculated according to Equation (1):

*η* = (*M_origin_ f_origin_* − *M_residue_f*_residue_)/*M_origin_f_origin_* = 1 − (*M_residue_f*_residue_/*M_origin_ f_origin_*) (1)

where *η* means the removal efficiency of heavy metals from MSWI fly ash (%); *M_origin_* and *M_residue_* means the dry basis quality of MSWI fly ash before and after treatment (kg); and *f_origin_* and *f*_residue_ means the heavy metal content in MSWI fly ash before and after treatment (mg/kg).

Heavy metal removal efficiency was investigated by examining the parameters of pH, current density, duration, and polarity switching frequency as well as EDTA dosage. The experimental arrangements are given in Table 1.

### 2.3. Total Heavy Metal Content

Microwave digestion refers to “The technical specification for soil environmental monitoring” (HJ/T 166-2004), and it is used to digest the fly ashes before and after the leaching. First, 0.2000 g of MSWI fly ash was weighed and added into the digestion tube; then, it was blended with 6 mL of HNO_3_ (69%), 2 mL of HCl (72%) and 2 mL of HF (48%). After covering lids, they were placed in the digestion tubes and treated by a microwave dissolver (Multiwave PRO, Anton Paar, Graz, Austria). After digesting according to the set temperature control procedure, they were cooled and filtered. Heavy metals such as Pb, Cu, Zn, Cd, Cr, Ni, As, and Ba in the filtrate were determined by ICP-OES.

### 2.4. Toxicity Characteristic Leaching Procedure

The toxicity leaching procedure specified in the standard of “Solid waste-extraction procedure for leaching toxicity—Sulphuric acid & nitric acid method” (HJT299-2007) was applied to assess the leaching toxicity of heavy metals. First, a 100 g sample was weighed and placed in an extraction bottle. According to the moisture content of the sample, the extraction agent was mixed evenly with the sample at a solid–liquid ratio of 10:1 (kg/L). Therein, an extraction agent was prepared from sulfuric acid and nitric acid with a mass ratio of 2:1 and a pH of 3.20 ± 0.05. After that, it was fixed on an inverted oscillation device after the lid is tightened, and it was overturned at a speed of 30 ± 2 r/min for 18 ± 2 h at 23 ± 2 °C. The concentration of heavy metals in the extracting solution was determined by ICP-OES.

### 2.5. Sequential Extraction Procedure

Heavy metals in different chemical forms were analyzed by selective extractors. According to the extractors, it can be extracted in three steps to minimize its mutual influence. (1) Soluble and Exchangeable: 40 mL 0.11 mol/L CH_3_COOH for 1.0000 g sample, oscillated for 16 h at room temperature. After being centrifuged for 20 min (4000 r/min), the liquid supernatant was filtered through a 0.45 μm membrane and retained for testing. Then, the remaining residue was added with 20 mL of deionized water, oscillated for 20 min, centrifuged for 20 min, and the liquid supernatant was discarded. (2) Reducible: using the remaining residue from the previous step as the subject, 40 mL 0.5 mol/L NH_2_OH·HCl was added; then, it wa oscillated at room temperature for 16 h, and the same operation as the previous step was repeated. (3) Oxidizable: 10 mL of H_2_O_2_ was slowly added to the residue in the centrifuge tube; then, it was covered and dissolved at room temperature for 1 h. After that, the solution was dissolved in a water bath for 1 h (85 °C); then, the lid was removed, and the water bath was heated until the solution was less than 3 mL. Afterwards, we added H_2_O_2_ 10 mL again and repeated the above operation. (4) Residual: the residue of the previous step was evaporated, transferred to the digestion tank, and digested.

### 2.6. Characterization

The sample was analyzed using an X-ray fluorescence spectrometer (XRF, UltimaIV, Rigaku, Tokyo, Japan). The mineral phase was determined by an X-ray diffractometer (XRD, Axios MAX, PANalytical B.V., Almelo, Netherlands). The micromorphology was observed by a field emission scanning electron microscope (FESEM, S-3400N, HITACHI, Tokyo, Japan). Cl^−^ was determined by an automatic potentiometric titration (888Titrando, Metrohm, Herisau, Switzerland). 

## 3. Results and Discussion

### 3.1. Characteristics of MSWI Fly Ash

As can be seen from Table 2, the dominating elements in MSWI fly ash are Ca, Cl, Si, S, Na, and K, accounting for 87.01 wt %. The high Ca content in MSWI fly ash is mainly caused by the excessive lime slurry injected into the flue gas to reduce the emission of acidic gases (SO_2_, HCl) [20]. Meanwhile, the plastic products, kitchen waste, and other chlorine components volatilize–condense during the incineration, so that there is a higher content of Cl (18.38 wt %) in MSWI fly ash in the form of soluble chlorine salts [21].

**Figure 2:**
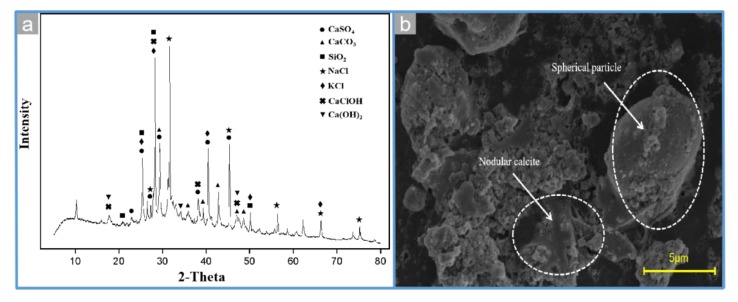
XRD patterns (**a**) and SEM images (**b**) of MSWI fly ash.

The mineralogical components of MSWI fly ash are shown in Figure 2a, and the main mineral phases of MSWI fly ash are KCl, NaCl, CaSO_4_, CaClOH, CaCO_3_, SiO_2_, and Ca(OH)_2_, which are consistent with the results of other research studies [22,23]. It indicates that MSWI fly ash is a mixture of chloride, calcium compounds, and silicate. However, the high content of amorphous substance enhances the background position of the XRD pattern, obscuring some crystal characteristic peaks, which could not reflect the mineral phase of the metal in the MSWI fly ash [24].

The micromorphology of MSWI fly ash is shown in Figure 2b. In Figure 2b, a large amount of amorphous substances is arranged loosely with a few spherical particles and floccule. Therein, non-mineral particles are dark in color, loose and porous in structure, with spherical particles of different particle sizes and nodular calcite (CaCO_3_) embedded on the surface, which is speculated to be secondary minerals congealed and generated in flue gas.

### 3.2. Characteristics of Heavy Metals in MSWI Fly Ash

A large amount of heavy metals is enriched in MSWI fly ash. Table 3 shows the heavy metal in MSWI fly ash and their leaching toxicity based on the extraction procedure of HJ/T 299-2007. It can be seen from Table 3 that the heavy metals in the MSWI fly ash are mostly Zn, Pb, Ba, and Cu, and their content reach to 3610.76 mg/kg, 1143.64 mg/kg, 1047.00 mg/kg, and 546.12 mg/kg, respectively. This means that the heavy metals in MSWI fly ash are not inert substances, showing a serious of extraction toxicity. Pb and Cd extracted reached 12.82 mg/L and 1.21 mg/L, which exceeded the threshold of GB 5085.3-2007 of 5 and 1 mg/L. Moreover, the leaching toxicity of Pb is more than double the standard threshold [25].

**Figure 3:**
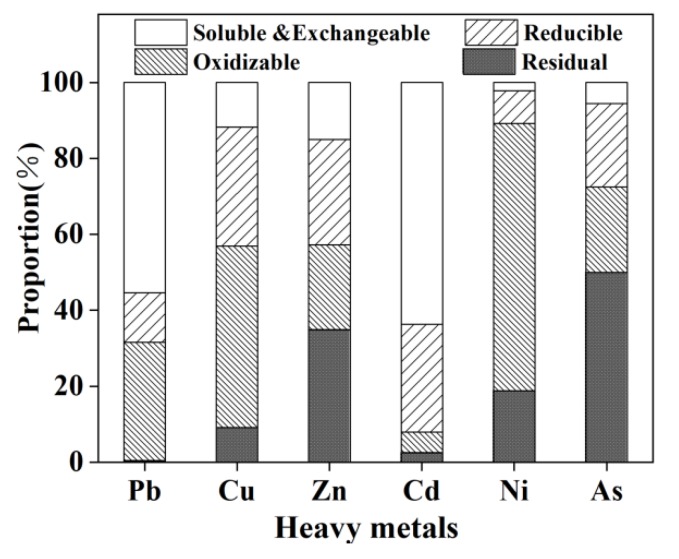
Heavy metal forms of MSWI fly ash.

Figure 3 shows the chemical distribution of different heavy metals in MSWI fly ash. The proportion of soluble to exchangeable forms of Cd is as high as 63.63%, and the proportion of residual form is relatively low, only 2.47%. This could be attributed to Cd being a kind of volatile heavy metal, which is easy to react with chlorine to form chloride in the incineration process. It finally adsorbs to MSWI fly ash in the condensation phase. Although its total content is only 132.48 mg/kg, its mobility and bioavailability in the environment are so high that it poses a serious risk to the environment [26]. The proportions of an oxidizable form of Pb, Cu, and As reach 55.34%, 47.82%, and 70.40%, respectively, which are relatively stable. However, it still faces the risk of leaching in a strong oxidizing environment. The residual form of Ni is 50.01%, which is a much lower risk to the environment when compared to other heavy metals. The difference of heavy metal chemical distribution is mainly related to its physicochemical characteristics (volatility, melting point, boiling point); it is hardly related to the total content of heavy metal, and these results are also consistent with other research studies [27]. 

### 3.3. Effect of pH

**Figure 4:**
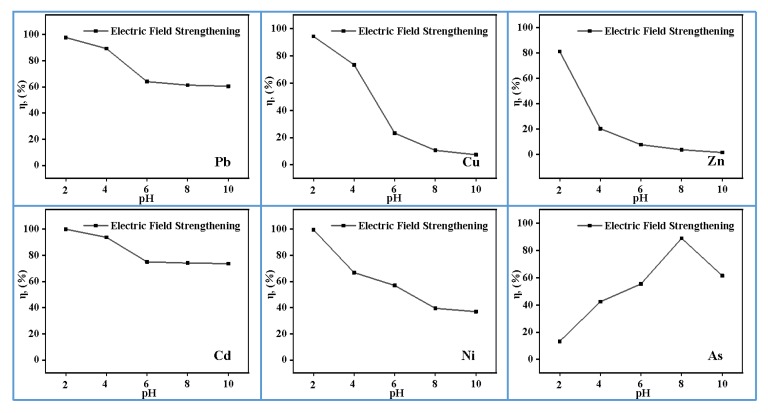
Effect of pH on heavy metals efficiency (0.5 mol/L EDTA, 25 mA/cm^2^, 4 h, 0 Hz).

Previous research studies show that pH is an important factor that affects the extraction of heavy metals from MSWI fly ash [28,29]. As shown in Figure 4, pH has a significant effect on the removal efficiency of heavy metals in MSWI fly ash. For Pb, Cu, Zn, Cd, and Ni, the removal efficiencies increase with the decrease of pH value, and reach the maximum at a pH of 2, which are 97.78%, 94.26%, 81.17%, 100%, and 99.59% respectively. The leaching characteristics of heavy metals in MSWI fly ash are bound up with the compounds formed. When the pH value changes, it would enhance/weaken the conversion of heavy metal to free ions in the system [30]. Under acidic conditions, heavy metals generally form metal oxides that are soluble in water, making the leaching characteristics more obvious. However, As in MSWI fly ash could only be effectively removed at a high pH value. It may be related to As existing as a stable complex of calcium or a transition metal of iron hydroxyl arsenate hydrate (M^2+^)_2_Fe_3_(AsO_4_)_3_(OH)_4_^−^ 10H_2_O. The complexes are degraded as the pH increases, where As is removed with the release of Ca^2+^ [31]. 

### 3.4. Effect of Current Density

**Figure 5:**
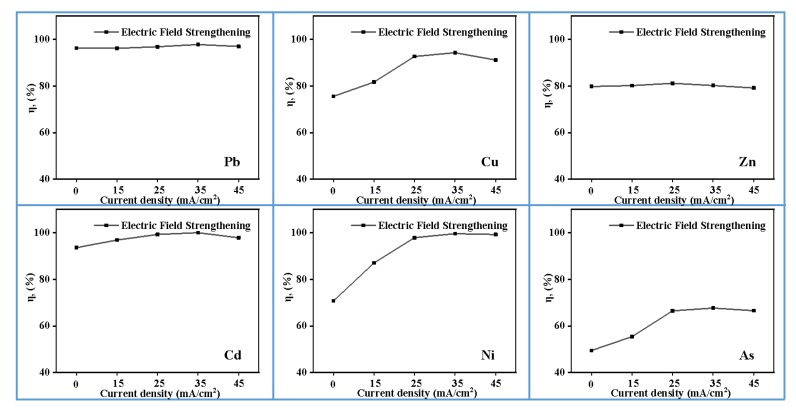
Effect of current density on heavy metals efficiency (0.5 mol/L EDTA, pH = 2, 4 h, 0 Hz).

The removal efficiency of heavy metals in MSWI fly ash, under different current densities, is shown in Figure 5. With the increasing of current density, the removal efficiency of Cu and Ni increase first and then plateau. When the current density is 35 mA/cm^2^ and pH is 2, the removal efficiency of Cu can reach 94.26%, while Ni in the MSWI fly ash is basically removed, about 99.59%. The removal efficiency of As increases from 49.48% to 67.69% when the current density increases from 0 to 35 mA/cm^2^. By changing the surface charge distribution on the face of MSWI fly ash and accelerating electron transfer in solution, the electric field can improve the removal efficiency of heavy metal [32]. However, an excess current density would cause energy waste and reduce the current efficiency.

### 3.5. Effect of Extraction Time

**Figure 6:**
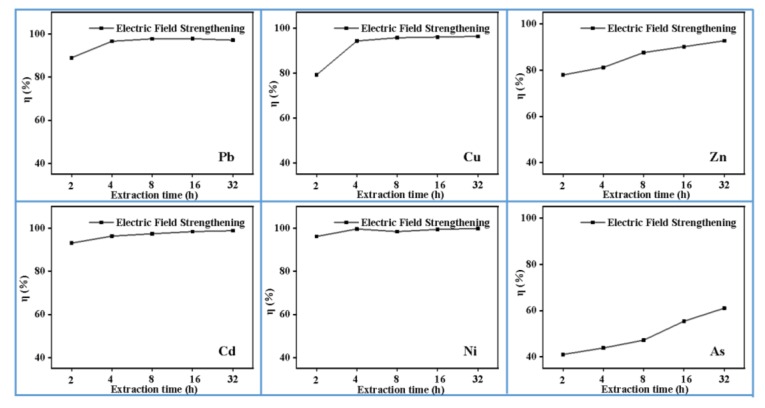
Effect of extraction time on heavy metals efficiency (0.5 mol/L EDTA, 35 mA/cm^2^, pH = 2, 0 Hz).

In order to investigate the effect of extraction time on the removal efficiency of heavy metals in MSWI fly ash, the time intervals were selected as 2 h, 4 h, 8 h, 16 h, and 32 h. Figure 6 shows that Cd and Ni can be removed quickly: more than 90% in 2 h. The removal efficiency curves of Pb and Cu increase first and then tend to flatten as the extraction time increases. When the extraction time is 4 h, the removal efficiency of Pb and Cu reach 97.78% and 96.01%, respectively. By contrast, Zn and As require a long extraction time, and the curves do not reach equilibrium, even extending the extraction time to 32 h. It would be related to the residual forms of Zn and As, since they are higher than 34.89% and 50.00%, making it difficult to transfer from the solid phase to the liquid phase. The interaction time between MSWI fly ash and agent affects the dissolution and diffusion of heavy metals directly. If the extraction time is too short, a sufficient dissolution cannot be guaranteed. By contrast, the disposal costs will rise.

### 3.6. Effect of Polarity Switching Frequency

**Figure 7:**
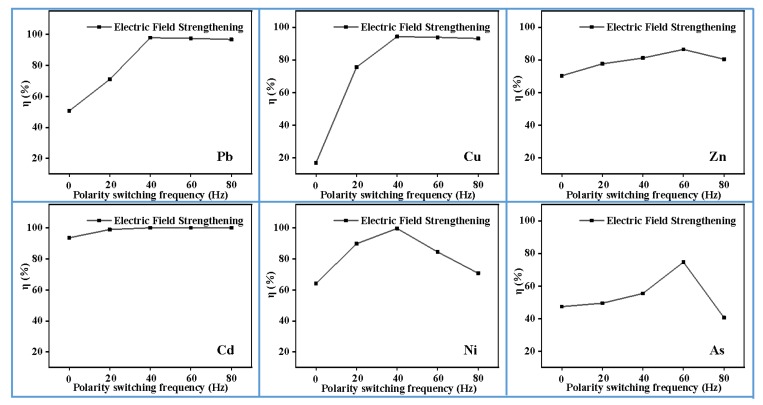
Effect of periodically switched polarity on heavy metals efficiency (0.5 mol/L EDTA, 35 mA/cm^2^, pH = 2, 2 h).

The removal efficiency of heavy metals in MSWI fly ash was investigated by periodically switching the polarity of cathode and anode. The results are shown in Figure 7. For Pb and Cu, the removal efficiency goes up with the increase of polarity switching frequency, and tends to plateau after 40 Hz. With the increase of polarity switching frequency, the removal efficiency of Ni and As increase first and then decrease, reaching their maximum at frequencies of 40 Hz and 60 Hz with values of 99.59% and 74.69%, respectively. Without polarity switching, during the extraction process, a large amount of Ca^2+^ and Mg^2+^ in the system would generate a dense oxide film adsorbing on the surface of the plate, which causes the plate to be passivated, thereby reducing the current efficiency [33]. This phenomenon was also reported by other researchers [34,35]. Polarity switching could alleviate this passivation. Under electric field, before the heavy metal ions are adsorbed on the electrodes, the motion direction will be changed due to the electrode switching. 

### 3.7. Characterization of Soluble Salts in MSWI Fly Ash During Extraction Process

**Figure 8:**
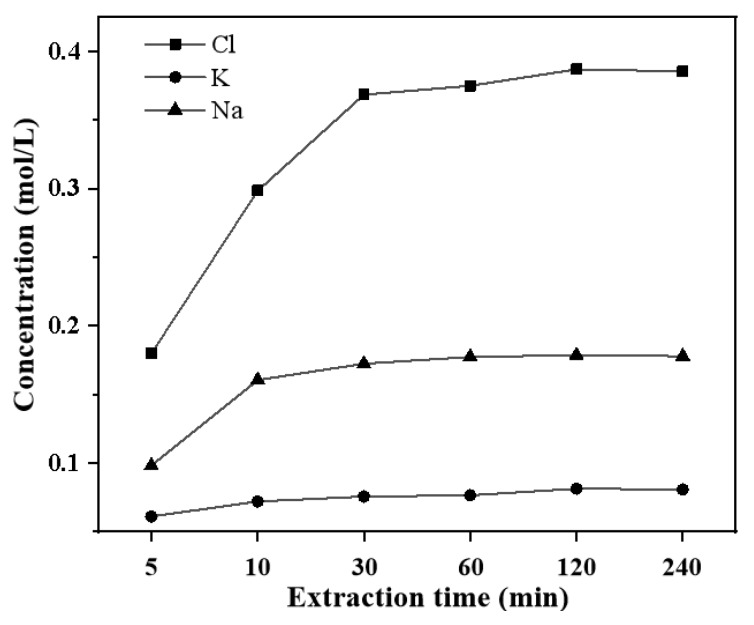
Composition changes in the extracting solution at different extraction times (mol/L).

It can be seen from Figure 8 that Cl^−^ can be quickly dissolved in solution and stabilized in about 10 to 30 min. The concentration of salt in MSWI fly ash during the extraction process was as follows: Cl^−^ > Na^+^ > K^+^, about 0.38 mol/L, 0.08 mol/L, and 0.18 mol/L respectively. The dissolution of Cl^−^, K^+^, and Na^+^ is related to the rapid dissolution of soluble components, such as NaCl, KCl, K_2_Ca(SO_4_)_2_·H_2_O, and the partial dissolution of insoluble aluminosilicate (K_2_O·Al_2_O_3_·6SiO_2_, Na_2_O·Al_2_O_3_·3SiO_2_·2H_2_O) and chloroaluminates (3CaO·Al_2_O_3_·CaCl_2_·10H_2_O). 

### 3.8. Characterization of the MSWI Fly Ash under Different Conditions

The chemical composition of the original samples and samples treated by the electric field are shown in Table 4. As shown in Table 4, compared with the original sample, the proportion of CaO, Cl, Na_2_O, and K_2_O in MSWI fly ash decreases obviously after treated by electric field. The mass of these four before and after the treatment decrease from 68.02 wt % to 28.82 wt %. This phenomenon indicates that the soluble salts in MSWI fly ash are effectively removed after being treated.

**Figure 9:**
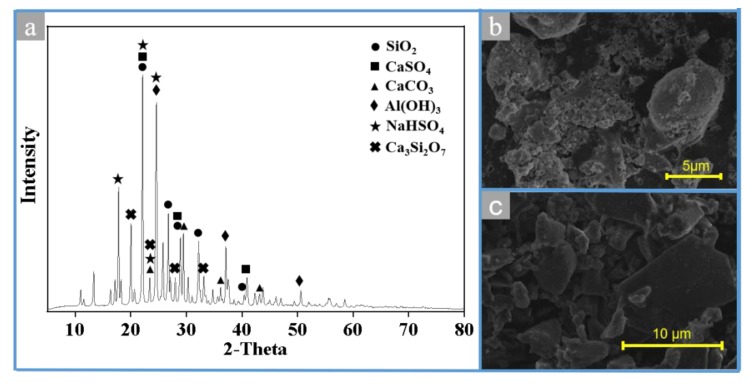
XRD patterns (**a**) and SEM images (**b**) and (**c**) of treated MSWI fly ash.

As can be seen from Figure 9a, the position of the diffraction peak of MSWI fly ash is significantly different before and after extraction. The primary mineral phases in the treated MSWI fly ash are SiO_2_, CaSO4, CaCO3, Ca_3_Si_2_O_7_, NaHSO_4_, and Al(OH)_3_. The diffraction peaks of KCl and NaCl in the sample treated by electric field disappear, while the diffraction peaks of SiO_2_ and CaSO_4_, which are hardly soluble in water, are present obviously. In addition, CaCO3 in MSWI fly ash reacts with H^+^ generated by the plate to produce CO_2_, which may explain the absence of Ca(OH)_2_ and CaClOH in treated MSWI fly ash.

Ca(OH)_2_ + CO_2_ → CaCO_3_ + H_2_O (2)

2CaClOH + CO_2_ → CaCl_2_+ CaCO_3_ + H_2_O (3)

SEM analysis of the samples is shown in Figure 9b,c. The distribution of the original sample is disordered, and a few spherical particles and floccule are overlapped (Figure 9b). As shown in Figure 9c, the irregular particles are significantly reduced. Most of the particles in treated MSWI fly ash are smooth clumps that are about less than 10 μm in size. These results show that the shape and size of MSWI fly ash particles can be changed by the electric field. 

It can be seen from Table 5 that the extraction toxicity of Pb and Cd in the original samples is 12.82 mg/L and 1.21 mg/L, respectively, exceeding the maximum permissible concentration of GB 5085.3-2007, 5, and 1 mg/L. After the treatment, various elements in the toxicity characteristic leaching procedure are reduced. The extraction toxicity of Pb and Cd are reduced by 94.58% and 100%, respectively, which are lower than the threshold specified in the standard. In addition, the extraction toxicity of most elements, such as Zn, Cd, Cr, Ni, and As in MSWI fly ash are lower than the detection limit.

**Figure 10:**
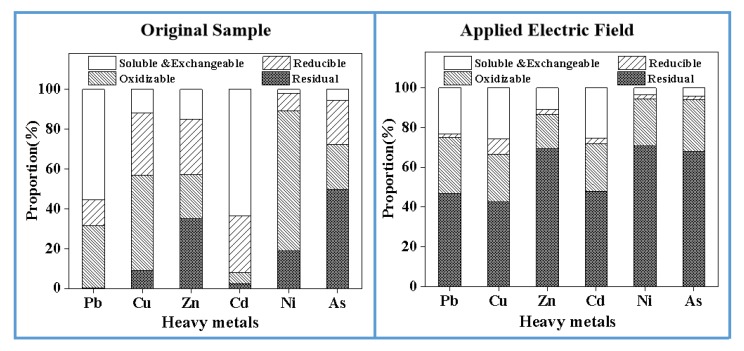
Heavy metal forms of treated MSWI fly ash.

As can be seen from Figure 10, after the treatment, compared with original samples, the residual form of Pb, Cu, Zn, Cd, Ni, and As are increased by 46.54%, 33.47%, 34.37%, 45.27%, 52.09%, and 18.02%, respectively. When the electric field was applied, other forms of heavy metals, except residual, are continuously removed. Therefore, the electric field can not only improve the extraction ability of heavy metals in the reaction system, but also increase the proportion of stable form of heavy metals, which reduces the harm to the environment.

### 3.9. The Main Mechanism of Pollutant Migration

**Figure 11:**
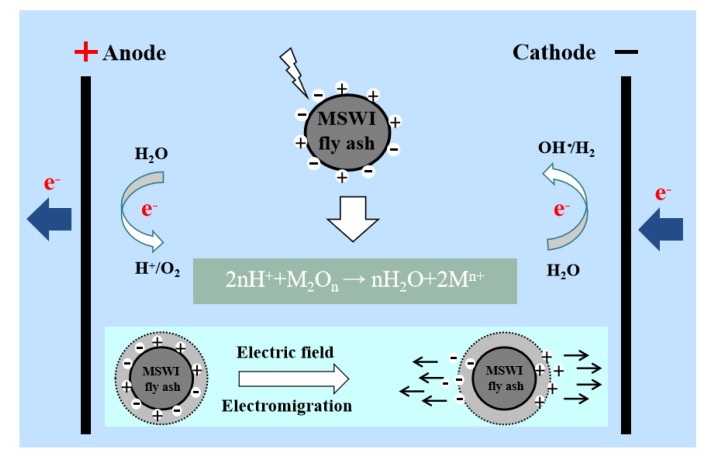
Electric field intensifies heavy metal in MSWI fly ash removal process.

The removal process of heavy metal in MSWI fly ash are illustrated in Figure 11. In the reaction system, MSWI fly ash is affected by the liquid diffusion and electrostatic attraction, and it is restricted by the pore solution, cations, and other substances. The surface charge polarity of MSWI fly ash will change under a certain pH, which be concerned with the reaction at the Equations (4) and (5). During this process, heavy metals release from the MSWI fly ash into the solution and react with the assistant agents to form a stable complex, as shown in Equations (6) and (7). The main reactions are as follows:

2H_2_O − 4e^−^ → O_2_↑+ 4H^+^ (4)

2H^+^ + 2e^−^ → H_2_↑ (5)

M^2+^+ H_2_Y^2−^→ MY^2−^+2H^+^ (6)

M^3+^+ H_2_Y^2−^→ MY^−^+2H^+^ (7)

## 4. Conclusions

The results of this research indicated that heavy metals could be efficiently removed by electric field enhanced washing. When the current density was 35 mA/cm^2^, polarity switching frequency was 40 Hz and EDTA dosage was 0.5 mol/L, at a pH value of 2 for 4 h, the removal efficiency of Cd, Ni, and Pb is up to 100.00%, 99.59%, and 97.78%, while Cu and Zn removal efficiency is higher than 85% and As is about 74.69%. The sequential extraction procedure showed that the residual forms of Pb, Cu, Zn, Cd, Ni, and As increased obviously. In addition, almost all of the K^+^, Na^+^, and Cl^−^ was removed at the same time as proven by XRD, XRF, and SEM. After the electric field-enhanced washing, the leaching toxicity of MSWI fly ash is greatly reduced when compared with the original samples, and metal leaching toxicity of all the elements is below all their thresholds. Therefore, electric field-enhanced washing has a perspective future in the removal of harmful substances from MSWI fly ash.

## Figures and Tables

**Table 1:** Experimental arrangement.

Factors	Levels
pH	2, 4, 6, 8, 10
Current density (mA/cm^2^)	0, 15, 25, 35, 45
Duration (h)	2, 4, 8, 16, 32
Switching frequency (Hz)	0, 20, 40, 60, 80

**Table 2:** Chemical composition of municipal solid waste incineration (MSWI) fly ash by X-ray fluorescence spectrometer (XRF) (wt %).

Compound	CaO	Cl	SiO_2_	SO_3_	Na_2_O	K_2_O	Al_2_O_3_	MgO	Fe_2_O_3_	Other
Original Sample	39.18	18.38	10.02	8.97	5.99	4.47	3.81	3.75	2.11	3.32

**Table 3:** Heavy metals content and their leaching toxicity in MSWI fly ash.

Heavy Metals		Pb	Cu	Zn	Cd	Cr	Ni	As	Ba
Original sample	Content (mg/kg)	1143.64	546.12	3610.76	132.48	176.65	19.35	123.12	1047.00
	Extraction toxicity (mg/L)	12.82	1.86	14.12	1.21	0.28	0.06	ND	2.77
GB 5085.3-2007 Threshold		5	100	100	1	15	5	5	100

Annotation: “ND” Not detected.

**Table 4:** Chemical composition of samples by XRF (wt.%).

Compound	CaO	Cl	SiO_2_	SO_3_	Na_2_O	K_2_O	Al_2_O_3_	MgO	Fe_2_O_3_	Other
Original samples	39.18	18.38	10.02	8.97	5.99	4.47	3.81	3.75	2.11	3.32
Electric field strengthening	20.35	1.65	32.71	1.04	3.11	3.71	20.52	0.83	6.61	9.47

**Table 5:** Extraction toxicity of treated MSWI fly ash (mg/L).

Heavy Metals	Pb	Zn	Cu	Cd	Cr	Ni	As	Ba
Original sample	12.82	1.86	14.12	1.21	0.28	0.06	ND	2.77
Electric field strengthening	1.78	ND	1.12	ND	ND	ND	ND	0.49
GB 5085.3-2007	5	100	100	1	15	5	5	100

Annotation: “ND” Not detected.
